# Phase Stability and Slag-Induced Destabilization in MnO_2_ and CeO_2_-Doped Calcia-Stabilized Zirconia

**DOI:** 10.3390/ma16227240

**Published:** 2023-11-20

**Authors:** Hwanseok Lee, Hee-Seon Lee, Seonghoon Kim, Kanghee Jo, Ilguk Jo, Heesoo Lee

**Affiliations:** 1 School of Materials Science and Engineering, Pusan National University, Busan 46241, Republic of Korea; hwanseok@pusan.ac.kr (H.L.); lhs9693@krict.re.kr (H.-S.L.); seonghoonkim@pusan.ac.kr (S.K.); jokanghee@pusan.ac.kr (K.J.); 2 Reliability Assessment Center, Korea Research Institute of Chemical Technology, Daejeon 34114, Republic of Korea; 3 Advanced Materials Engineering, Dong-Eui University, Busan 47340, Republic of Korea

**Keywords:** calcia-stabilized zirconia, slag, submerged entry nozzle, immersion resistance, phase stability, stabilizing agents

## Abstract

MnO_2_ and CeO_2_ were doped to improve the corrosion resistance of CSZ (calcia-stabilized zirconia), and we studied the phase formation, mechanical properties, and corrosion resistance by molten mold flux. The volume fraction of the monoclinic phase gradually decreased as the amount of MnO_2_ doping increased. The splitting phenomenon of the t(101) peak was observed in 2Mn_CSZ, and in 4Mn_CSZ, it was completely split, forming a cubic phase. The relative density increased and the monoclinic phase decreased as the doping amount increased, leading to an increase in Vickers hardness and flexural strength. However, in 3Mn_CSZ and 4Mn_CSZ, where cubic phase formation occurred, the tetragonal phase decreased, leading to a reduction in these properties. MnO_2_-doped CSZ exhibited a larger fraction of the monoclinic phase compared to the original CSZ after the corrosion test, indicating worsened corrosion resistance. These results are attributed to the predominant presence of Mn^3+^ and Mn^2+^ forms, rather than the Mn^4+^ form, which has a smaller basicity difference with SiO_2_, and due to the low melting point. The monoclinic phase fraction decreased as the doping amount of CeO_2_ increased in CeO_2_-doped CSZ, but the rate of decrease was lower compared to MnO_2_-doped CSZ. The monoclinic phase decreased as the doping amount increased, but the Vickers hardness and flexural strength showed a decreasing trend due to the low relative density. The destabilization behavior of Ca in SEM-EDS images before and after corrosion was difficult to identify due to the presence of Ca in the slag, and the destabilization behavior of Ce due to slag after corrosion was not observed. In the XRD data of the specimen surface after the corrosion test, the fraction of the monoclinic phase increased compared to before the test but showed a lower monoclinic phase fraction compared to CSZ. It is believed that CeO_2_ has superior corrosion resistance compared to CaO because Ce predominantly exists in the form of Ce^4+^, which has a smaller difference in basicity within the zirconia lattice.

## 1. Introduction

The submerged entry nozzle (SEN) is a functional refractory material that plays a crucial role in the final stage of continuous casting, situated between the tundish and the mold [1]. It prevents contact between the molten metal and air while ensuring a steady flow of molten metal into the mold and stabilizing the flow of the molten metal [2]. The SEN must possess excellent thermal shock resistance to withstand rapid temperature changes and sufficient strength to bear the pressure of the molten metal. Additionally, it requires high corrosion resistance and wear resistance against molten metal and slag for prolonged usage [3,4]. The body of the SEN primarily utilizes Al_2_O_3_-C-based refractories, but in the areas that contact slag, ZrO_2_-C-based refractories are used due to their superior corrosion resistance and abrasion resistance [5]. In ZrO_2_-C-based refractories, ZrO_2_ is chemically stable, resistant to forming low-melting-point compounds, and has excellent thermal spalling resistance. Graphite minimizes thermal shock by providing excellent thermal conductivity and reduces contact and reactions between slag and the refractory material due to its low wettability with slag [6]. However, ZrO_2_-C-based refractories are known to be eroded by slag [7]. It is commonly understood that erosion occurs due to the flow at the interface between slag and molten metal [8,9]. The interface between slag and molten metal fluctuates, causing the ZrO_2_-C refractory to alternately come into contact with the molten metal and slag. When the molten metal rises, graphite dissolves into the molten metal, exposing the surface of ZrO_2_. When the molten metal descends, ZrO_2_ is exposed to the slag without graphite, and it is known that ZrO_2_ dissolves into the slag, leading to erosion. The dissolution of ZrO_2_ by the slag is known to be the rate-determining step, given the rapid dissolution of graphite into the molten metal in this erosion mechanism [10]. However, this mechanism explains erosion at the interface between slag and molten metal, but it does not explain erosion that occurs further up from the interface. Therefore, Hauck and Potschke proposed a localized corrosion mechanism due to interfacial tension gradients [11]. This mechanism suggests that slag covers ZrO_2_ due to surface tension, forming a slag film and eroding ZrO_2_. In this manner, the submerged entry nozzle is explained by complex erosion mechanisms, and ultimately, since the erosion rate of ZrO_2_ determines the life of the submerged entry nozzle, research is being conducted to reduce the erosion rate of ZrO_2_ and to decrease the graphite content [12]. However, the reduction in graphite content causes a lower thermal conductivity in ZrO_2_-C refractories, which can lead to a decrease in thermal shock resistance. Therefore, there is a need to secure thermal shock resistance through the improvement of the mechanical properties of zirconia.

Partially stabilized zirconia (PSZ), which exhibits excellent thermal shock resistance due to transformation toughening as cubic or tetragonal phases transform to the monoclinic phase, is used for submerged entry nozzles [13,14]. PSZ is obtained by stabilizing high-temperature phases at room temperature by adding stabilizers such as MgO, CaO, and Y_2_O_3_. Primarily, CaO-stabilized zirconia (CSZ) is used for submerged entry nozzles [15]. However, CSZ has issues with corrosion caused by slag, and the destabilization of zirconia due to reactions with slag is known to be the main cause. SiO_2_ present in the slag reacts with CaO to form a low-melting-point glass, and it is known to transform cubic or tetragonal phases of zirconia into the monoclinic phase, breaking up the zirconia grains [16,17]. To improve the durability of submerged entry nozzles, it is necessary to address the destabilization of zirconia caused by slag, and progress is being made in enhancing corrosion resistance through co-doping.

Corrosion of refractories by slag is related to the basicity and viscosity of the slag, and Hirata et al. have presented an experimental equation to predict the corrosion rate of ceramic materials by slag [18].

(1) log⁡∆D=0.18log⁡∆B−0.55log⁡η+C

where ∆D is the depth of corrosion (μm), ∆B is the basicity gap between slag and ceramics, η is the viscosity of the slag, and *C* is a constant that varies depending on the corrosion conditions. The basicity gap can be calculated through the ∆*B* parameter proposed by Morinaga et al., estimated as follows [19]:

(2) $$B=\Sigma n_{i}B_{i}$$

(3) $$B_{i}=\frac{\left(\frac{{\left(r_{i}+1.40\right)}^{2}}{Z_{i}\times 2}\right)-0.405}{1.023}$$

where $n_{i}$ represents the fraction of cation i for all cations, and $r_{i}$ and $Z_{i}$ represent the ionic radius and charge of cation i, respectively. This parameter can represent the basicity of completely molten oxides, assuming that the bond between cations and anions in the slag is ionic. From this equation, we can deduce that the basicity increases as the ionic radius increases and the charge decreases, and to reduce reactivity with SiO_2_, it is necessary to dope with acidic elements.

In this study, we aimed to enhance the corrosion resistance of CaO-stabilized zirconia (CSZ) by doping it with acidic CeO_2_ and MnO_2_ and analyzing the resultant mechanical properties. XRD analysis was performed to investigate the changes in crystal structure due to doping, including variations in monoclinic phase fraction and peak shifts/corrosion tests were conducted to assess the corrosion resistance of CeO_2_ and MnO_2_-doped CSZ and analyze the associated destabilization behavior.

## 2. Experimental Procedures

**Figure 1:**
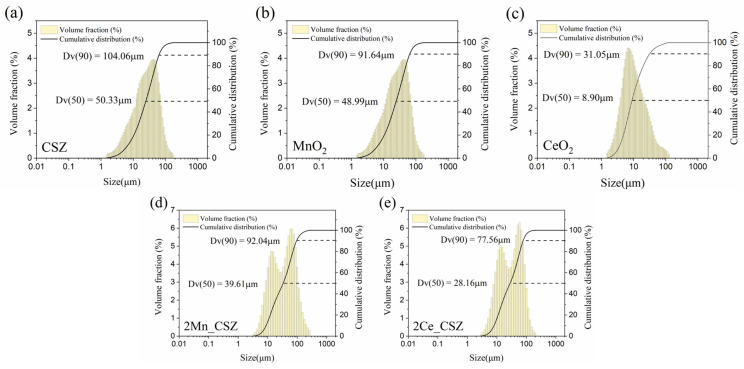
Particle size distribution of (**a**) CSZ powder, (**b**) MnO_2_ powder, (**c**) CeO_2_ powder, (**d**) 2Mn_CSZ powder after calcination and (**e**) 2Ce_CSZ powder after calcination.

**Figure 2:**
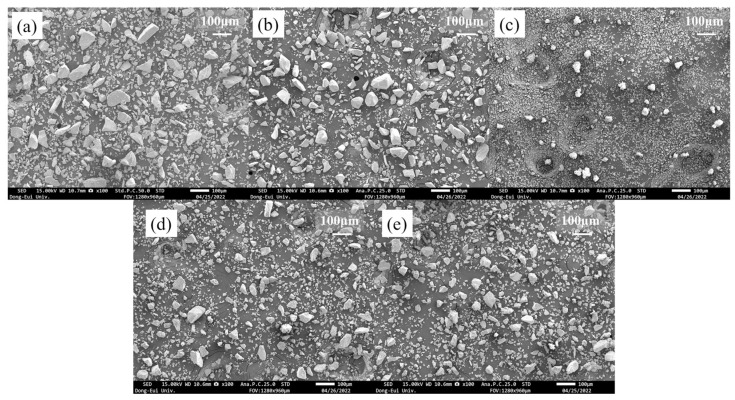
(**a**) CSZ powder, (**b**) MnO_2_ powder, (**c**) CeO_2_ powder, (**d**) 2Mn_CSZ powder after calcination, and (**e**) 2Ce_CSZ powder after calcination.

CaO-stabilized zirconia (CSZ) is a commercial refractory powder, and its chemical composition is listed in Table 1. MnO_2_ (Junsei Chemical Co., Ltd., Kanto, Japan, 90%) powder and CeO_2_ (Kanto Chemical Co., Inc., Tokyo, Japan, 99.99%) were used as dopants. The particle size distribution characteristics and SEM images of the powders are shown in Figure 1 and Figure 2. MnO_2_-doped CSZ and CeO_2_-doped CSZ were prepared as shown in Table 2. MnO_2_ and CeO_2_ were introduced into CSZ powders by the mechanical mixing method. The mixed powders were ball milled in ethanol for 24 h. Mixed solutions were then dried at 100 °C using a dry oven, calcinated for 24 h using a dry oven, and then calcined at 1200 °C for 2 h in a box-type electric furnace. Powder was uniaxially pressed at 3 ton/m^2^ to produce a 20 mm disk specimen, and the green body was sintered at 5 °C/min to various temperatures (1300–1600 °C) for 4 h. The bar-type specimens (3.5 mm × 4.5 mm × 36 mm) were uniaxially pressed at 3.2 ton/m^2^ in a steel die and heated up to 1600 °C with a heating rate of 5 °C/min for 4 h.

A corrosion test was performed using slag powder, and the composition of the slag powder is indicated in Table 3. 0.7 g of the slag powder was formed into pellets using a cylindrical 15 mm mold with a pressure of 1 ton/m^2^. The slag pellets were then placed on sintered zirconia specimens and heat-treated in a box furnace at 1550 °C for 10 h in an air atmosphere.

The densities of sintered pellets were determined using the Archimedes’ method, which involved immersing the samples in distilled water. X-ray diffraction (XRD) patterns of the specimens were collected at room temperature using a step scan procedure (2θ = 10–90°, with a step interval of 0.02°) and Cu-Kα radiation on a Rigaku Ultima-IV XRD instrument (Rigaku Corporation, Tokyo, Japan) at the Converging Materials Core Facility. The XRD data obtained for phase analysis was analyzed using Rigaku SmartLab Studio II software version 4.3.101.0.

Monoclinic phase was determined from the integrated intensity of the X-ray diffraction scan based on ISO 5803 [20].

(4) $$X=\frac{I{\left(\overset{-}{1}11\right)}_{m}+I{\left(111\right)}_{m}}{I{\left(\overset{-}{1}11\right)}_{m}+I{\left(111\right)}_{M}+I{\left(101\right)}_{t}}$$

(5) $$X=\frac{I{\left(\overset{-}{1}11\right)}_{m}+I{\left(111\right)}_{m}}{I{\left(\overset{-}{1}11\right)}_{m}+I{\left(111\right)}_{M}+I_{ttc}}$$

Formular (4) is for a two-phase system (monoclinic and tetragonal phases), and Formular (5) is for multi-phase system (a mixture of monoclinic, tetragonal, and cubic). X is an integrated intensity ratio. Where $I{\left(\overset{-}{1}11\right)}_{m}$ and $I{\left(111\right)}_{m}$ refer to the integral intensity of the X-ray diffraction pattern of monoclinic. The $I{\left(101\right)}_{t}$ refers to the integral intensity from the (101) plane of tetragonal and $I_{ttc}$ is total integrated intensity of tetragonal phase (101) and cubic phase (111) reflection. Calculate the volume fraction of the monoclinic phase using Formular (6):

(6) $$f_{m}=\frac{PX}{1+\left(P-1\right)X}$$

$f_{m}$ is the volume fraction of the monoclinic phase and the P is the intensity factor. In the monoclinic-tetragonal ZrO_2_ system, P=1.219 is used, while in the multiphase system, P=1.265 is used.

A nano indenter (FISCHERSCOPE, HM2000, Sindelfingen, Germany) was used to measure the Vickers hardness. Flexural strength was measured with the three-point bending method using test specimens measuring 3.5 mm × 4.5 mm × 36 mm. The SEM images of the samples were obtained by using a scanning electron microscope (JEOL Ltd., JSM-IT800, Tokyo, Japan).

## 3. Results and Discussion

Figure 1 presents the particle size analysis (PSA) data of the powders. CSZ and MnO_2_ consisted of coarser particles with average diameters of 50.33 μm and 48.99 μm, respectively, while CeO_2_ comprised finer particles with an average diameter of 8.90 μm. This trend is well illustrated in Figure 2, where CSZ and MnO_2_ exhibit non-uniform, coarse single particles. Figure 1d,e presents the PSA data of 2Mn_CSZ and 2Ce_CSZ powders, which were wet ball milled and calcined at 1200 °C, demonstrating a reduction in average diameter compared to the original CSZ. Figure 2d,e displays smaller particle sizes compared to the original CSZ. Additionally, the average particle diameter of 2Ce_CSZ, which is doped with the relatively finer CeO_2_, was 28.16 μm, smaller than that of 2Mn_CSZ at 39.61 μm.

**Figure 3:**
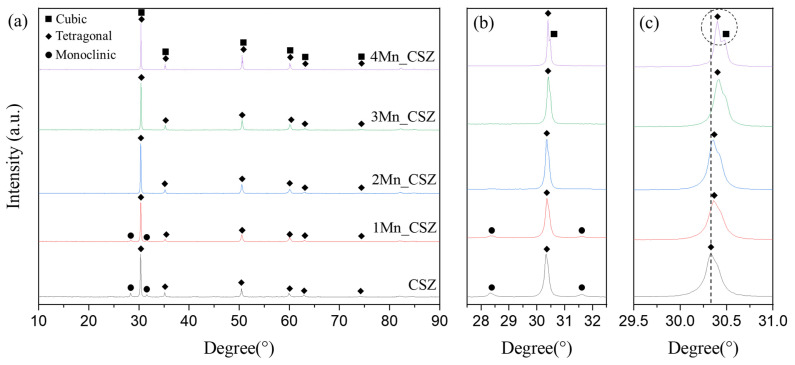
(**a**) XRD diffraction patterns (**b**) m ($11\overset{-}{1}$) and m (111) peaks and (**c**) t(101) and C (111) peaks of MnO_2_-doped CSZ.

Figure 3 shows the XRD patterns of MnO_2_-doped CSZ sintered at 1600 °C for 4 h. In CSZ, both the monoclinic phase (ICSD 98-006-0900, space group of *P1 21/c 1*) and the tetragonal phase (ICSD 98-007-0014, space group of *P 42/nmc*) were observed, with the monoclinic volume fraction reaching 33.7%. As shown in Figure 3b, the monoclinic peak gradually decreased with the increase in MnO_2_ doping amount. The monoclinic volume fraction was 19.7% in 1Mn_CSZ, while the monoclinic phase was not observed in 3Mn_CSZ and 4Mn_CSZ. In 2Mn_CSZ, the phenomenon of peak splitting at the t(101) peak was observed, and as the doping amount increased, the peak splitting became more distinct. In 4Mn_CSZ, complete splitting was confirmed, and the formation of a cubic phase (ICSD 98-010-5553, space group *Fm-3m*) was verified through Figure 2c, with the cubic phase fractions in 3Mn_CSZ and 4Mn_CSZ being determined to be 4.6% and 9.1%, respectively (Table 4). In addition, a higher angle shift was observed with the increase in doping amount, suggesting a change in the valence state of Mn during doping. Clavel et al. reported that when the content of Mn is less than 1.4%, Mn exists mainly in the form of Mn^2+^ (96 Å) in zirconia, which is larger than Zr^4+^ (84 Å). However, as the Mn content increases, Mn mainly exists as Mn^3+^ (64.5 Å), which has a smaller ionic radius than Zr^4+^, inducing lattice distortion [21]. Thus, the doped Mn mainly forms a substitutional solid solution as Mn^3+^, creating oxygen vacancies and stabilizing the monoclinic phase into tetragonal and cubic phases [22].

**Figure 4:**
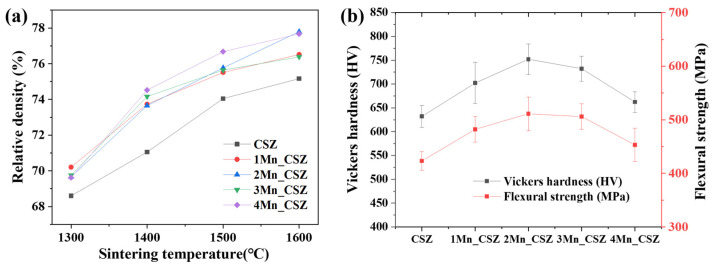
(**a**) Relative density vs. sintering temperature, and (**b**) Vickers hardness and flexural strength of MnO_2_-doped CSZ.

Figure 4a shows the graph of the relative density changes of MnO_2_-doped CSZ with temperature. The relative density appearing in the 70% range is believed to be attributed to the larger particle size of the zirconia refractory powder, as shown in Figure 1a, resulting in incomplete densification. In the SEM images of the specimen sintered at 1600 °C (Supplementary Figure S1), it was difficult to observe microstructural changes due to density changes because the overall density was low. It was observed that the relative density increased with the doping of MnO_2_, while CSZ displayed a relative density of 75.16% at 1600 °C, 4Mn-doped CSZ exhibited a higher relative density of 77.68%. MnO_2_, having a low melting point of 535 °C, acted as an additive, enhancing the relative density [23]. Figure 4b presents the Vickers hardness and flexural strength of MnO_2_-doped CSZ. As MnO_2_ was doped, the Vickers hardness steadily increased from 632.12 Hv (CSZ) to 752.12 Hv (2Mn_CSZ), and the flexural strength increased from 423.25 MPa (CSZ) to 511.23 MPa (2Mn_CSZ). These results are attributed to the increase in relative density and the decrease in monoclinic phase fraction due to MnO_2_ doping [24]. However, as the doping amount of MnO_2_ increased further, the mechanical properties decreased. The Vickers hardness dropped to 732.26 Hv (3Mn_CSZ) and 662.35 Hv (4Mn_CSZ), and the flexural strength decreased to 505.89 MPa (3Mn_CSZ) and 453.25 MPa (4Mn_CSZ). Despite the increase in relative density, the decrease in mechanical properties is considered to be related to the formation of the cubic phase. The formation of the cubic phase reduces the tetragonal phase, diminishing the transformation toughening effect arising from the martensitic phase transformation from tetragonal to monoclinic phases [25].

**Figure 5:**
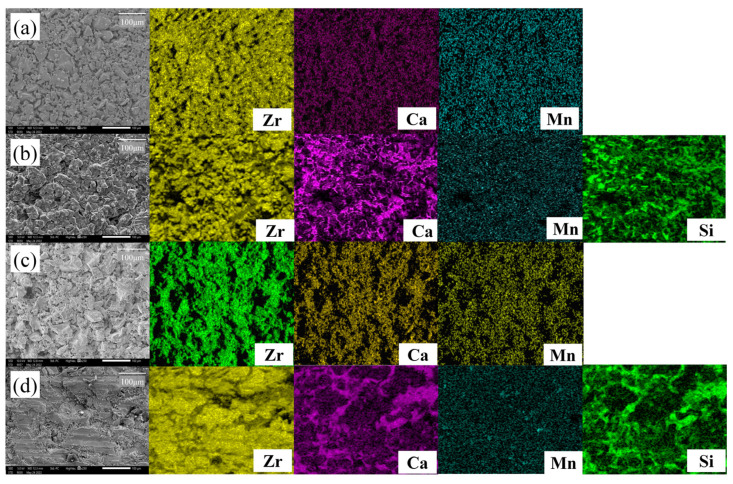
SEM-EDS surface images of 2Mn_CSZ (**a**) before corrosion, and (**b**) after corrosion and SEM-EDS corrosion cross-sectional images of 2Mn_CSZ (**c**) before corrosion, and (**d**) after corrosion test (same magnification).

Figure 5 shows SEM-EDS images of 2Mn_CSZ magnified 250 times before and after corrosion, where the specimen sintered at 1600 °C was subjected to corrosion in slag at 1550 °C for 10 h. In the pre-corrosion surface image of 2Mn_CSZ (Figure 5a), it is apparent that full densification has not occurred due to the coarse particle size, and EDS scanning revealed that Ca and Mn were uniformly dispersed without agglomeration. Figure 5b shows the surface of 2Mn_CSZ after corrosion, and the EDS scans of Ca and Si indicate that residues of slag remain on the surface. The presence of slag residues made it difficult to observe the destabilization behavior due to the leaching of Mn and Ca from 2Mn_CSZ caused by the slag. The post-corrosion cross-sectional SEM-EDS images of 2Mn_CSZ (Figure 5d) reveal that the slag has penetrated the interior due to the high porosity of the specimen, making it impossible to evaluate the internal corrosion resistance through the assessment of the penetration depth of the slag.

**Figure 6:**
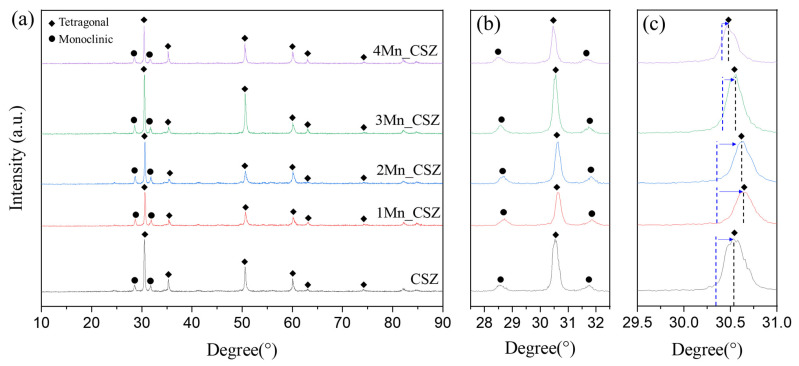
(**a**) XRD diffraction patterns, (**b**) m (11$\overset{-}{1}$) and m (111) peaks, and (**c**) t(101) peaks of MnO_2-_doped CSZ after corrosion test.

Figure 6 shows the XRD data of the MnO_2_-doped CSZ specimens’ surface after corrosion. After the corrosion, only the tetragonal and monoclinic peaks were observed in MnO_2_-doped CSZ, while no slag peaks were detected. This is due to the formation of a glass phase during the rapid cooling process following the corrosion test [26]. In 4Mn_CSZ, although a cubic peak was initially observed, no cubic phase was detected after corrosion. As indicated in Table 5, the cubic volume fraction decreased from 9.1% before corrosion to 0% after corrosion, which is considered to be a phase transition caused by destabilization. In Figure 6c, it can be seen that the CSZ has shifted to a higher angle after corrosion (from 30.34° to 30.52°), indicating that Ca^2+^ ions, which are larger than Zr^4+^ ions, have leached out from the lattice due to reactions with the slag. Before the corrosion test, the MnO_2_-doped CSZ showed a low-angle shift due to the doping of smaller Mn^3+^ and Mn^4+^ ions, positioning the t(101) at a lower angle compared to CSZ. In particular, the 1Mn_CSZ exhibited the largest shift from 30.36° before corrosion to 30.66° after corrosion. However, after the corrosion test, the t(101) position was at a higher angle than that in the undoped case. These results corresponded with the monoclinic phase fraction results, with 1Mn_CSZ showing the highest monoclinic phase fraction of 42.7% after corrosion. As the amount of MnO_2_ doping increased, the angle shift to a higher angle after corrosion decreased, with the 4Mn_CSZ shifting from 30.40° before corrosion to 30.46° after corrosion. These results suggest that Mn, being smaller than the zirconia ions, has also leached out from the zirconia lattice due to destabilization, resulting in a combined effect of a lower angle shift due to Mn leaching and a higher angle shift due to Ca leaching. All specimens with Mn doping showed a higher fraction of the monoclinic phase compared to the original CSZ, indicating a decrease in corrosion resistance. As mentioned earlier, the corrosion of stabilized zirconia by slag is primarily due to the leaching of stabilizers by SiO_2_ [16,17]. The $B_{i}$ values of SiO_2_ and CaO calculated using Equation (3) are 0.470 and 1.613, respectively. Since SiO_2_ and CaO not only form a glass phase but also have a large difference in basicity, a significant reaction can be expected. The $B_{i}$ values for MnO_2_, Mn_2_O_3_, and MnO are 0.536, 0.796, and 1.563, respectively. Although the $B_{i}$ of MnO_2_ does not differ significantly from that of SiO_2_, it is expected that the reaction will be minimal. However, since Mn in the zirconia lattice mainly exists in the form of Mn^3+^, there is a difference in basicity, leading to potential instability. Additionally, the melting points of MnO_2_ and Mn_2_O_3_ are 535 °C and 940 °C, respectively, which are significantly lower than the 2572 °C of CaO, suggesting that leaching into the slag would be easier.

**Figure 7:**
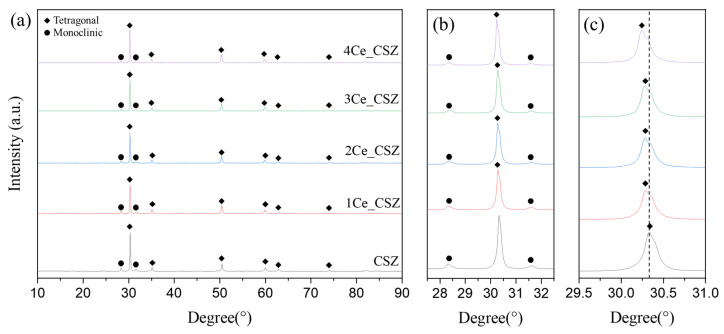
(**a**) XRD diffraction patterns, (**b**) m (11$\overset{-}{1}$) and m (111) peaks, and (**c**) t(101) of CeO_2_-doped CSZ.

Figure 7 presents the XRD patterns of CeO_2_-doped CSZ, which was sintered at 1600 °C for 4 h. Both monoclinic and tetragonal phases were observed in the CeO_2_-doped CSZ. As seen in Figure 7b, the monoclinic peak gradually decreased with the increase of CeO_2_ doping, resulting in a decrease in the monoclinic volume fraction from 29.5% in 1Ce_CSZ to 21.8% in 4Ce_CSZ (Table 6). In Figure 7c, a lower angle shift occurred with the increase in doping amount, indicating that the lattice expanded due to the doping of Ce^4+^ (97 Å), which is larger than Zr^4+^ (84 Å) [27]. When compared to MnO_2_-doped CSZ, the rate of decrease in the monoclinic phase was lower. This is because when MnO_2_ is doped, it mainly forms a substitutional solid solution in the form of Mn^3+^ (64.5 Å), creating oxygen vacancies to maintain charge neutrality, leading to a significant stabilization effect due to the formation of these vacancies [28]. On the other hand, when CeO_2_ is doped, both Ce^4+^ and Ce^3+^ exist, predominantly in the form of Ce^4+^. Thus, stabilization primarily occurs due to lattice stress caused by the ionic radius difference between Zr^4+^ and Ce^4+^, resulting in a weaker effect on stabilizing the tetragonal phase compared to MnO_2_ [29].

**Figure 8:**
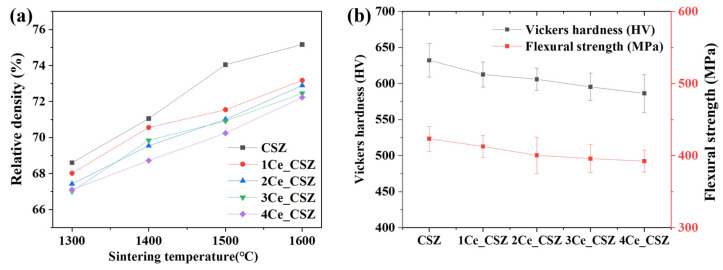
(**a**) Relative density vs. sintering temperature, and (**b**) Vickers hardness and flexural strength of CeO_2_-doped CSZ.

Figure 8a illustrates the graph of relative density changes in CeO_2_-doped CSZ, indicating a trend of decreasing relative density with increasing amounts of CeO_2_ doping. The SEM images of the specimen sintered at 1600 °C (Supplementary Figure S2) show that, due to the overall low density, it was challenging to observe microstructural changes associated with density changes. The relative density gradually decreased from 75.1% (CSZ) to 72.23% (4Ce_CSZ) at 1600 °C, and this result is attributed to the fact that CeO_2_ generates a low sinterability system [30]. Figure 8b illustrates the Vickers hardness and flexural strength of CeO_2_-doped CSZ. The Vickers hardness decreased from 612.57 Hv (1Ce_CSZ) to 586.35 Hv (4Ce_CSZ), and the flexural strength also reduced from 412.63 MPa (1Ce_CSZ) to 392.22 MPa. Even though an increase in tetragonal phase was observed with an increase in CeO_2_ doping, the sinterability deteriorated, leading to a decrease in mechanical properties.

**Figure 9:**
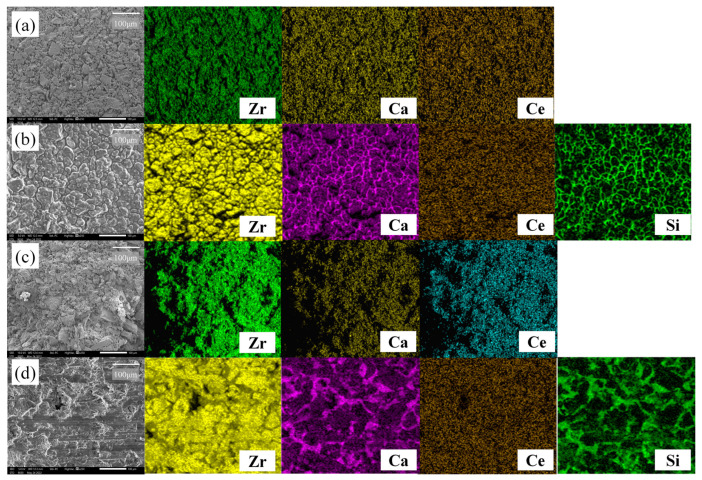
SEM-EDS surface images of 2Ce_CSZ (**a**) before corrosion, and (**b**) after corrosion and SEM-EDS corrosion cross-sectional images of 2Ce_CSZ (**c**) before corrosion, and (**d**) after corrosion test (same magnification).

Figure 9 shows the SEM-EDS images of 2Ce_CSZ magnified 250 times before and after corrosion. In the pre-corrosion surface image of 2Ce_CSZ, Figure 9a, it is observable that complete densification has not occurred due to the coarse particle size, and the EDS scan confirmed that Ca and Ce were evenly dispersed without agglomeration. After corrosion, the surface of 2Ce_CSZ shows signs of deterioration due to the slag. The EDS scans of Ca and Si reveal remnants of slag left on the surface, particularly concentrated near the grain boundaries that formed post-corrosion. Discerning the destabilization behavior of Ca was challenging due to the presence of Ca in the slag, and no destabilization behavior of Ce due to slag post-corrosion was observed. Figure 9d presents the cross-sectional SEM-EDS images of 2Ce_CSZ after corrosion, where the infiltration of slag into the interior due to high porosity can be seen.

**Figure 10:**
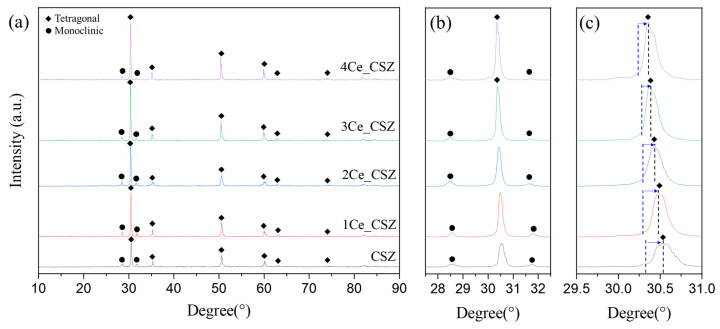
(**a**) XRD diffraction patterns, (**b**) m (11$\overset{-}{1}$) and m (111) peaks, and (**c**) t(101) of CeO_2_-doped CSZ after corrosion.

Figure 10 shows the surface XRD data of CeO_2_-doped CSZ after corrosion. Similar to MnO_2_-doped CSZ, no slag peaks were observed in CeO_2_-doped CSZ post-corrosion; instead, tetragonal and monoclinic peaks were present. After corrosion, the monoclinic phase fraction calculated through the integrated intensity of X-ray diffraction increased due to destabilization, though it remained lower than that of CSZ (Table 7). Additionally, as the doping amount of CeO_2_ increased, the monoclinic phase fraction further decreased. In Figure 10c, the t(101) peak exhibited a high angle shift due to destabilization, with less shift occurring as the Ce doping amount increased. The calculated $B_{i}$ value for CeO_2_ using Equation (3) is 0.702, not significantly different from the $B_{i}$ value of SiO_2_ (0.470), indicating a small basicity gap. Consequently, it is inferred that CeO_2_-doped CSZ demonstrates superior corrosion resistance compared to CaO, and unlike MnO_2_-doped CSZ, Ce is not easily leached by the slag, allowing the tetragonal phase to be maintained.

## 4. Conclusions

We doped CSZ with MnO_2_ and CeO_2_, respectively, to enhance its corrosion resistance and investigated the mechanical properties and slag-induced destabilization behavior. The monoclinic phase volume fraction decreased from 33.7% (CSZ) to 0% (3Mn_CSZ, 4Mn_CSZ) as the doping amount of MnO_2_ increased. A splitting phenomenon of the t(101) peak was observed in 2Mn_CSZ, and a complete splitting resulting in a cubic phase occurred in 4Mn_CSZ. It was noted that the relative density increased with MnO_2_ doping, showing a relative density of 77.68% in 4Mn-doped CSZ compared to 75.16% in CSZ at 1600 °C. The increase in relative density and decrease in monoclinic phase fraction with MnO_2_ doping resulted in an increase in Vickers hardness and flexural strength, though these properties decreased in 3Mn_CSZ and 4Mn_CSZ, where a cubic phase was formed. The monoclinic phase fraction of CSZ increased from 33.7% to 39.4% after the corrosion test. The MnO_2_-doped CSZ had a higher monoclinic phase fraction compared to CSZ after the corrosion test, resulting in reduced corrosion resistance, and the monoclinic phase fraction of 4Mn_CSZ increased from 0% to 42.4%. These results are attributed to the predominant existence of Mn in the forms of Mn^3+^ and Mn^2+^, rather than Mn^4+^, and their low melting points due to the small basicity gap with SiO_2_. With CeO_2_-doped CSZ, an increase in the CeO_2_-doping amount led to a decrease in the monoclinic phase fraction, though at a lower rate than in MnO_2_-doped CSZ. This is because Ce in the zirconia lattice mainly exists in the form of Ce^4+^, and the stabilization of the tetragonal phase is mainly due to the lattice stress caused by the ionic radius difference between Zr^4+^ and Ce^4+^, resulting in a less significant stabilizing effect than MnO_2_. CeO_2_-doped CSZ showed a lower relative density compared to CSZ due to its low sinterability, leading to a decrease in Vickers hardness and flexural strength. Pre- and post-corrosion SEM-EDS images indicated that the destabilizing behavior of Ca was challenging to observe due to the presence of Ca in the slag, and no post-corrosion destabilizing behavior of Ce due to slag was observed. The XRD data from the specimen surface post-corrosion exhibited a lower monoclinic phase fraction compared to CSZ, decreasing progressively with increased CeO_2_ doping. These results suggest that CeO_2_ primarily exists in the form of Ce^4+^ within the zirconia lattice, demonstrating superior slag resistance compared to CaO due to the small basicity gap. For further study, we aim to conduct research on simultaneously doping MnO_2_ and CeO_2_ to combine the high phase stability of MnO_2_ for improved thermal shock resistance and enhanced corrosion resistance from CeO_2_.

## Figures and Tables

**Table 1:** Chemical composition of CSZ powder (mol. %).

ZrO_2_	CaO	HfO_2_	SiO_2_	Al_2_O_3_	TiO_2_
81.818	13.707	1.739	1.894	0.324	0.517

**Table 2:** Composition of the CSZ specimens with different amounts of MnO_2_ and CeO_2_.

Compound	Composition		
	MnO_2_ (mol%)	CeO_2_ (mol%)	CSZ
1Mn_CSZ	1	-	Balance
2Mn_CSZ	2	-	
3Mn_CSZ	3	-	
4Mn_CSZ	4	-	
1Ce_CSZ	-	1	
2Ce_CSZ	-	2	
3Ce_CSZ	-	3	
4Ce_CSZ	-	4	

**Table 3:** Chemical composition of slag powder (mol. %).

Slag Powder	SiO_2_	CaO	Na_2_O	MgO	Al_2_O_3_
Composition (mol. %)	35.500	35.919	23.897	2.940	1.743

**Table 4:** Monoclinic volume fraction of MnO_2_-doped CSZ.

Specimen	Volume Fraction (%)		
	Monoclinic (V_m_)	Tetragonal (V_t_)	Cubic (V_c_)
CSZ	33.7	66.3	-
1Mn_CSZ	19.7	80.3	-
2Mn_CSZ	2.8	97.2	-
3Mn_CSz	-	95.4	4.6
4Mn_CSZ	-	90.9	9.1

**Table 5:** Monoclinic volume fraction of MnO_2_-doped CSZ after corrosion test.

Specimen	Volume Fraction (%)		
	Monoclinic (V_m_)	Tetragonal (V_t_)	Cubic (V_c_)
CSZ	33.7 → 39.4	66.3 → 33.7	-
1Mn_CSZ	19.7 → 42.7	80.3 → 57.3	-
2Mn_CSZ	2.8 → 42.5	91.2 → 57.5	-
3Mn_CSz	- → 40.0	95.4 → 60.0	4.6 → -
4Mn_CSZ	- → 42.4	90.9 → 57.6	9.1 → -

**Table 6:** Monoclinic volume fraction of CeO_2_-doped CSZ after corrosion test.

c	Volume Fraction (%)		
	Monoclinic (V_m_)	Tetragonal (V_t_)	Cubic (V_c_)
CSZ	33.7	66.3	-
1Ce_CSZ	29.5	70.5	-
2Ce_CSZ	27.9	72.1	-
3Ce_CSz	24.6	75.4	-
4Ce_CSZ	21.8	78.2	-

**Table 7:** Monoclinic volume fraction of CeO_2_-doped CSZ after corrosion.

Specimen	Volume Fraction (%)		
	Monoclinic (V_m_)	Tetragonal (V_t_)	Cubic (V_c_)
CSZ	33.7 → 39.4	66.3 → 60.6	-
1Ce_CSZ	29.5 → 33.5	70.5 → 66.5	-
2Ce_CSZ	27.9 → 31.2	72.1 → 68.8	-
3Ce_CSz	24.6 → 27.5	75.4 → 72.5	-
4Ce_CSZ	21.8 → 24.8	78.2 →75.2	-

## Data Availability

The data and analysis in this study are available on request from the corresponding authors.

## Supplementary Materials

The following supporting information can be downloaded at: https://www.mdpi.com/article/10.3390/ma16227240/s1, Figure S1: SEM surface images of (a) CSZ, (b) 1Mn_CSZ, (c) 2Mn_CSZ, (d) 3Mn_CSZ and (e) 4Mn_CSZ sintered at 1600 °C. Figure S2: SEM surface images of (a) CSZ, (b) 1Ce_CSZ, (c) 2Ce_CSZ, (d) 3Ce_CSZ and (e) 4Ce_CSZ sintered at 1600 °C.
