# Supplementary figures and images for: Phase Stability and Slag-Induced Destabilization in MnO2 and CeO2-Doped Calcia-Stabilized Zirconia
Source: Materials (Basel). 2023 Nov 20;16(22):7240. doi: 10.3390/ma16227240 (PMC10673052; doi:10.3390/ma16227240)

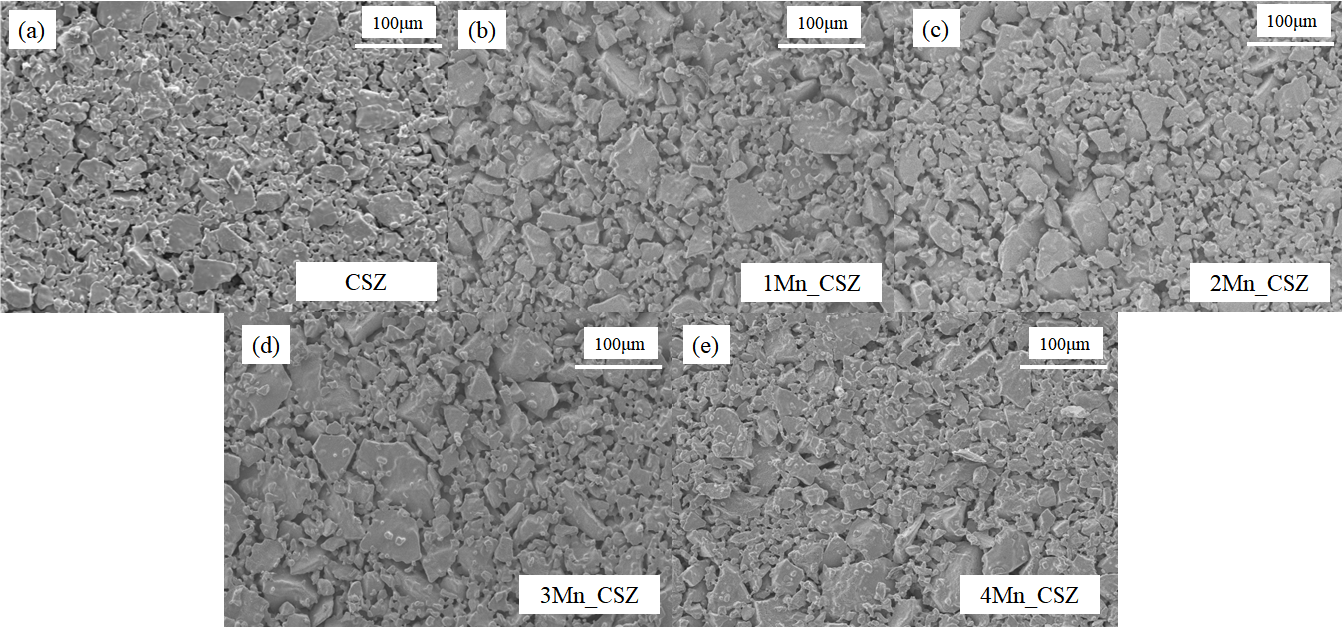

Supplement: Supplementary file 1 [file materials-16-07240-s001.zip › Supplementary Figure S1.tif]

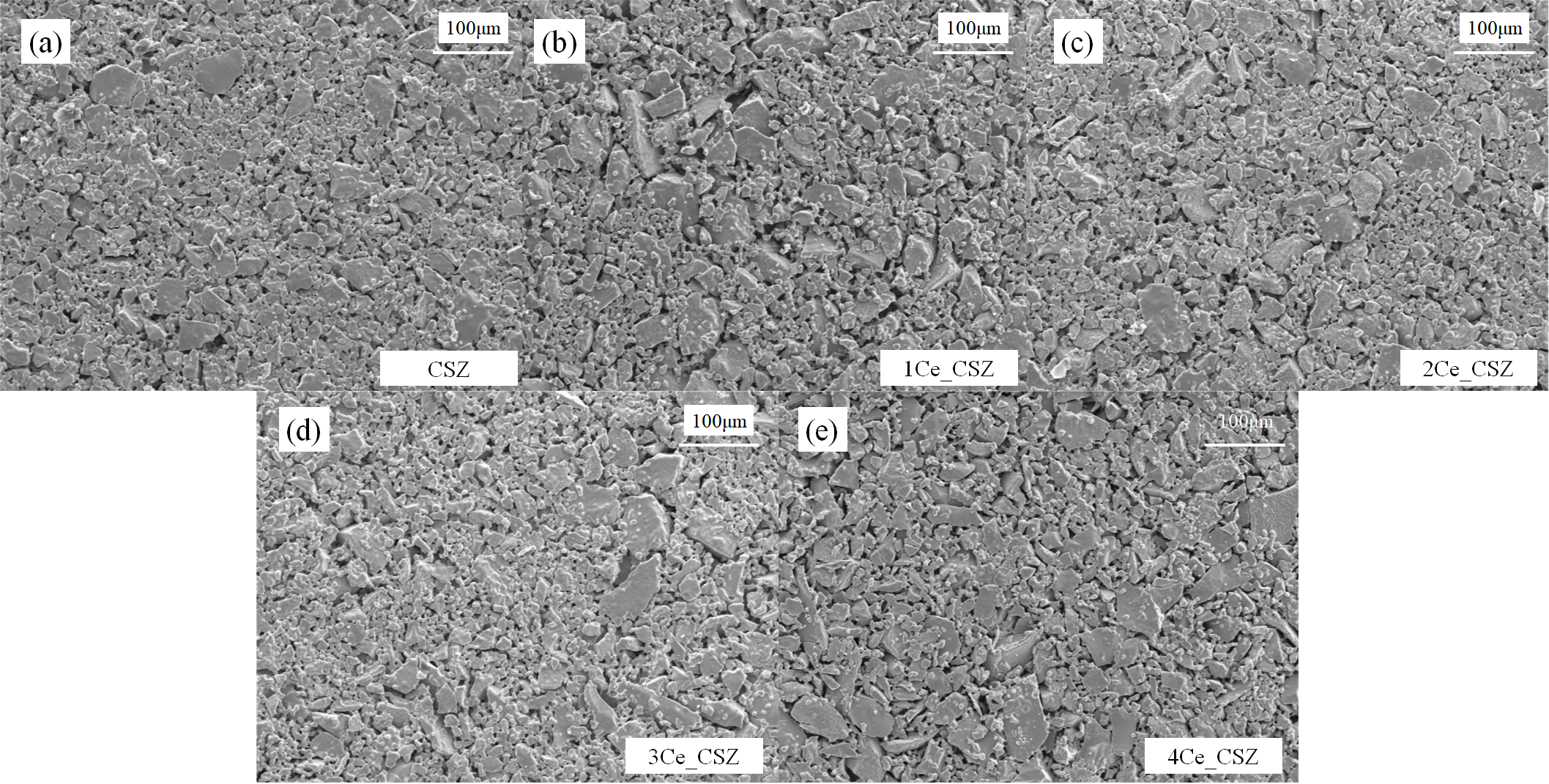

Supplement: Supplementary file 1 [file materials-16-07240-s001.zip › Supplementary Figure S2.tif]
